# Supplementary material for: A Critical View of the Application of the APEX Software (Aqueous Photochemistry of Environmentally-Occurring Xenobiotics) to Predict Photoreaction Kinetics in Surface Freshwaters
Source: Molecules. 2019 Dec 18;25(1):9. doi: 10.3390/molecules25010009 (PMC7017383; doi:10.3390/molecules25010009)
Supplement: Supplementary file 1 [file molecules-25-00009-s001.zip › 3.2.4_gcc-4.4.0.zip!/3.2.4_gcc-4.4.0/3.2.4_gcc-4.4.0/doc/octave/html/interpreter/Floating_002dPoint-Conversions.html]

Floating-Point Conversions - Untitled


Next: Other Output Conversions,
Previous: Integer Conversions,
Up: C-Style I/O Functions


---

#### 14.2.9 Floating-Point Conversions

This section discusses the conversion specifications for floating-point
numbers: the %f, %e, %E, %g, and %G
conversions.

The %f conversion prints its argument in fixed-point notation,
producing output of the form
[`-`]ddd`.`ddd,
where the number of digits following the decimal point is controlled
by the precision you specify.

The %e conversion prints its argument in exponential notation,
producing output of the form
[`-`]d`.`ddd`e`[`+`|`-`]dd.
Again, the number of digits following the decimal point is controlled by
the precision. The exponent always contains at least two digits. The
%E conversion is similar but the exponent is marked with the letter
E instead of e.

The %g and %G conversions print the argument in the style
of %e or %E (respectively) if the exponent would be less
than -4 or greater than or equal to the precision; otherwise they use the
%f style. Trailing zeros are removed from the fractional portion
of the result and a decimal-point character appears only if it is
followed by a digit.

The following flags can be used to modify the behavior:

-: Left-justify the result in the field. Normally the result is right-justified. +: Always include a plus or minus sign in the result. : If the result doesn't start with a plus or minus sign, prefix it with a space instead. Since the + flag ensures that the result includes a sign, this flag is ignored if you supply both of them. #: Specifies that the result should always include a decimal point, even if no digits follow it. For the %g and %G conversions, this also forces trailing zeros after the decimal point to be left in place where they would otherwise be removed. 0: Pad the field with zeros instead of spaces; the zeros are placed after any sign. This flag is ignored if the - flag is also specified.

The precision specifies how many digits follow the decimal-point
character for the %f, %e, and %E conversions. For
these conversions, the default precision is `6`. If the precision
is explicitly `0`, this suppresses the decimal point character
entirely. For the %g and %G conversions, the precision
specifies how many significant digits to print. Significant digits are
the first digit before the decimal point, and all the digits after it.
If the precision is `0` or not specified for %g or
%G, it is treated like a value of `1`. If the value being
printed cannot be expressed precisely in the specified number of digits,
the value is rounded to the nearest number that fits.
